# Complement proteins in unexpected places: why we should be excited, not concerned!

**DOI:** 10.12688/f1000research.21690.1

**Published:** 2020-02-26

**Authors:** Berhane Ghebrehiwet

**Affiliations:** 1 Department of Medicine, Stony Brook University, Stony Brook, New York, 11794-0001, USA

**Keywords:** Complosome, intracellular complement

## Abstract

The complement system consists of more than 30 plasma as well as cell surface proteins that together constitute a major arm of the immune system. The long-held belief is that most of the complement components are synthesized by hepatocytes in the liver and then secreted into the blood. However, there is also substantial evidence that several if not all of the complement proteins are synthesized extrahepatically by a wide range of cell types, including polymorphonuclear leukocytes, monocytes, macrophages, dendritic cells, lymphocytes, epithelial cells, fibroblasts, and neuronal cells. However, despite the proven evidence that complement proteins indeed could be synthesized non-hepatic cells and even found in unexpected places, the recent finding that certain complement proteins could be activated in intracellular spaces nonetheless has opened up a new debate. In fact, some in the field unfortunately seem to be in favor of rejecting this notion rather vehemently on the untenable and myopic grounds that complement proteins *could not* be found in intracellular compartments despite evidence to the contrary. Therefore, this opinion article is meant to remind colleagues in the field that new discoveries with the potential to shift established functional paradigms should be encouraged and celebrated even if, at first glance, they seem to defy the odds.

## Introduction

For any student of the biological sciences—or any of the sciences for that matter—nothing is more thrilling than the identification of a new molecule or a new pathway, since either potentially heralds the unraveling of a heretofore unrecorded or unanticipated biological function. However, such excitement sometimes is dampened by a few polemicists in the given field who reject or challenge the veracity of a new discovery outright on the basis of a *post hoc ergo propter hoc* logic. This logic stipulates that the location and function of each protein in our system are *already* known, *ergo* the presence of any protein outside its established locale must be an anomaly. This logic is, of course, erroneous because it is based on the false premise that plasma proteins are found in plasma and intracellular proteins are found only inside the cell and not anywhere else. This, in fact, is not true. Indeed, members of all classes of proteins—cytoskeletal components, secreted growth factors, glycolytic enzymes, kinases, transcription factors, chaperones, transmembrane proteins, and extracellular matrix proteins—have been identified in cellular compartments other than their conventional sites of action ^1, 2^. Furthermore, even highly conserved mitochondrial proteins are now being found performing novel extramitochondrial functions in unexpected extramitochondrial sites. This would suggest that mitochondrial proteins not only possess novel mechanisms for protein export to other sites ^2^ but also may have distinct novel roles in each compartment—be it intracellular or extracellular. This would suggest that each has the potential to participate in a variety of health and disease processes. Similarly, if proteins that should not be expected inside the cell are found inside the cell, then there must be a logical and functional reason why the protein is there, and it is for us to dig down and investigate.

Rational controversy or healthy disagreement in any field of science is part of the maturation and solidification process and therefore should be encouraged. But hasty, illogical, and dogmatic critique should be avoided lest it discourage those who have the courage to look outside the box to show us that novel paradigms could be unraveled if we look, without bias, deeper in unexpected places. Indeed, that is what research should be all about. Yet instead of challenging or encouraging these bright and daring scientists to generate more data to prove their cases, we often consciously or unconsciously discourage them by rejecting their proposed premise outright without a leg to stand on. Indeed, rejection and controversy are not new to any branch of science, least of all the complement field. In fact, some of the best ideas and discoveries, including Einstein’s, have been mistakenly rejected ^3, 4^. However, a hasty and reckless rejection unfortunately has far-reaching professional and personal consequences. First, the same people who reject the novel concept unfortunately also happen to be the ones who review and reject the manuscripts submitted for publication. Second—and psychologically more damaging—is the fact that these skeptics or other like-minded individuals also sit on many grant review panels and literally trash such a proposal when submitted for funding to agencies such as the National Institutes of Health. The list of brilliant ideas that were rejected and consequently never saw the light of day is too long to provide here. Fortunately, some discoveries have also stood the test of time, largely due to the undying courage and self-confidence of the proponents. However, it should be mentioned that the kind of unfair rejection and the inability to procure funding are, in fact, what drive many a bright and aspiring young scientist to abandon his or her project—and sometimes even the very science they love—in disgust. Therefore, if we are to advance real science, a rush to harsh judgment should be avoided; instead, we should challenge the primary investigator to generate more data to prove his or her premise. In fact, we know from experience that not every novelty turns out to be right. But once it is solidly proven wrong, no one else has to fall into the same trap again. This kind of attitude could only encourage the young flag-bearers or even the seasoned scientists to be more productive and inventive.

## Complement proteins in unexpected places: novel concepts and shifting paradigms

The complement system, like any of the other biological sciences, is not new to controversy. Since the discovery of complement by Buchner in the late 19th century ^5^, the description of each of the more than 30 proteins that make up the complement system and the three independent pathways has had its share of formidable challenges and controversies. No one has suffered the “slings and arrows” of painful rejection more than the brilliant scientist, Louis Pillemer ^6, 7^. Although he was the genius behind the discovery of the properdin (alternative) pathway—in addition to many other seminal contributions to the field—his discovery was not well received by his contemporaries, whose rationale was that a complement pathway (at that time, only the “C1 pathway” was recognized) that does not require antibody for activation would be impossible to comprehend ^6– 8^. Even in this day and age of sophisticated technology where one can prove or disprove a particular scientific question overnight, individual scientists find it easier to reject a novel concept outright if it does not satisfy their preconceived notion of what the concept should be than to give the proponent a chance to prove the concept.

One of the most exciting discoveries of the past few years is the finding of key complement proteins such as C3 and C5 in the intracellular compartment of T cells. These proteins in turn can be activated intracellularly and cross-talk with the inflammasome. According to this novel concept, T cells contain endosomal and lysosomal pools of C3, which can be processed into biologically active C3a and C3b by the T cell cathepsin ^9– 11^. These active fragments in turn serve the cell for homeostatic survival, whereas translocation of these fragments may induce autocrine pro-inflammatory cytokine production ^9– 11^. However, although this novel function of C3 and C5 in homeostatic survival of T cells is very exciting, one has to address the concern of those who might say, “If this is the mechanism of T-cell survival, why doesn’t genetic deficiency of the entire C3 gene in mice or humans lead to T-cell failure?”. In fact, many animal disease models seem to show that genetic deficiency in C3 does not exacerbate the disease process, suggesting that the mechanism might be different ^12, 13^.

The presence of complement proteins inside the cell, in and of itself, is not a novel observation since the existence of intracellular C proteins inside both immune and non-immune cells has been documented before ^14– 18^. In fact, almost all types of cells are known to synthesize complement proteins, some of which are stored inside the cell. However, it is the discovery of the existence of a crosstalk between intracellular complement and the inflammasome that has ignited excitement in the field. This novel concept has a name: it is called the “complosome” ^9^! This discovery was not appreciated by a few in the field, some of whom expressed their open disagreement at the XXVI International Complement Workshop, even though a whole session of the workshop (held in Kanazawa City, Japan, in 2016) was assigned to complosome. But it is surprising and difficult to understand how the veracity and relevance of intracellular complement proteins are still being questioned despite the robust evidence that has been accumulated to date. Not surprisingly, the significance of intracellular complement was again discussed under the title “What do we mean by intracellular complement?” at the 17th European Meeting on Complement in Human Disease (EMCHD) held recently in Madrid. However, the fact that it was brought up again for discussion supports the tenet that the function of intracellular complement has *finally* sparked the interest it deserves since the questions that were being asked were legitimate and appropriate. Therefore, we anticipate that this new area of research will remain a mainstay for the near future, as it would help us understand how intracellular complement proteins cross-talk with molecules of the inflammasome and potentially other intracellular proteins in both health and disease.

As mentioned above, several laboratories have shown the presence of complement or complement-like proteins inside the cell ^14, 16, 17^. The human neutrophils, for example, contain intracellular stores of CR1, C3, FB, and properdin ^14^. Upon neutrophil activation, the surface CR1 increases from a few thousand to 30,000 to 50,000 per cell. In addition, FB, properdin, and C3 are released from stores to trigger a local inflammatory process. Whether this process involves a traditional cascade reaction or simple proteolysis to release functionally active fragments such as C3a or Ba is not yet known. Similarly, the presence of C1q or C1q-like proteins has been shown in cell lysates ^15, 18, 19^, although the structure of the chains, especially the A-chain, appears to be different than that of the A-chain of C1q purified from plasma ^18^. More interestingly, the presence of C1q globular domain (gC1qD) inside the cell is well documented ^20^. The C1q molecule is a member of the tumor necrosis factor alpha (TNFα) superfamily of proteins, which includes adiponectin, and contains a “gC1qD” domain—a highly conserved domain—found in most of the TNFα superfamily of proteins ^21^. Furthermore, both the receptor for the gC1qD, called gC1qR, and the receptor for the collagen domain, called cC1qR or calreticulin (CRT), are also found in many compartments inside the cell ^22, 23^. Although the exact function of these proteins has yet to be elucidated, it is plausible to assume that an interaction between intracellular C1q or gC1qD and its receptor gC1qR, similar to those described for C3 ^24, 25^, exists inside the cell and is potentially involved in either apoptosis or cell proliferation. Therefore, understanding of the interaction between these intracellular complement proteins and other molecules may reveal unexpected functions with the potential to unlock some biological mysteries of health and disease.

Finally, as the discussants at the recent EMCHD suggested, the structure as well as the function of the plasma complement proteins and their intracellular homologues might indeed differ because of post-translational modifications or enzymatic cleavages. Like those of gC1qD, the structure and function of intracellular C3 and C5 may have been designed for purposes other than complement activation. Therefore, it would not be unreasonable to assume that different versions or structures of the same molecule may exist intracellularly for the purpose of fulfilling currently unrecognized functions. But the existence of complement proteins inside the cell should not be in dispute anymore. Therefore, it behooves us to encourage and support the pioneers in this area who had the foresight to think outside the box and unravel novel locations and functions of complement proteins. Like many earlier discoveries that—despite the initial antagonism—have enriched the complement field, the discovery of complement proteins inside the cell should now open a new chapter in the ever-growing and fascinating field that is complement. Discovery in science is like a river in that “the water that you see is the last of what has gone but is the first of what is to come”. Therefore, we should hope that we are able to see, for the best is yet to come!

## Conclusions

Controversy in science is not unique. Most of the great biomedical discoveries that we now take for granted were initially met with outright skepticism and rejection. Indeed, it would have stayed as such had it not been for the brave few who fought back to prove that their observations or discoveries were indeed correct. Some of the brave and self-confident ones had to go to extraordinary lengths: Barry Marshall drank broth containing *Helicobacter pylori*–—when the animal studies did not work—to provide evidence that this bacterium was indeed the cause of peptic ulcer. Although in the end he was recognized for his pioneering work by winning the Nobel Prize in 2005, he would not have had to go through such a risky experiment had his colleagues believed him in the first place. From Barry Marshall to Peyton Rous, the discoverer of the virus that now bears his name, many a brilliant scientist had to suffer unfair rejection before he or she was finally recognized. However, in the words attributed to Winston Churchill: “Success is not final and failure is not fatal, it is the courage to continue that counts”. And despite all the ephemeral fuss, continue we should! It may take a while before we figure out the significance of complement proteins inside the cell, but it is time we give this nascent area of complosome the applause and recognition it greatly deserves.
